# Out‐of‐Pocket Costs and Surprise Billing in Otolaryngology: A National Database Analysis

**DOI:** 10.1002/ohn.70035

**Published:** 2025-10-13

**Authors:** Nicholas R. Lenze, Chamila D. Perera, Karan R. Chhabra, John W. Scott, Raj C. Dedhia, Michael J. Brenner

**Affiliations:** ^1^ Department of Otolaryngology–Head and Neck Surgery University of Michigan Medical School Ann Arbor Michigan USA; ^2^ Department of Biostatistics University of Michigan Ann Arbor Michigan USA; ^3^ Department of Surgery New York University Grossman School of Medicine New York New York USA; ^4^ Division of Bariatric & General Surgery NYC Health+Hospitals/Bellevue New York New York USA; ^5^ Department of Surgery University of Washington School of Medicine Seattle Washington USA; ^6^ Department of Otorhinolaryngology University of Pennsylvania School of Medicine Philadelphia Pennsylvania USA

**Keywords:** ambulatory surgical procedures, database, fees and charges, health expenditures, health policy, healthcare costs, insurance coverage, MarketScan, otolaryngology, surprise billing

## Abstract

**Objective:**

To evaluate out‐of‐pocket (OOP) costs and surprise billing (unexpected charges from out‐of‐network providers) in otolaryngology.

**Study Design:**

Retrospective cohort study.

**Setting:**

National commercial claims database.

**Methods:**

Merative MarketScan database was queried for commercially insured patients aged 18 to 64 who underwent any of six otolaryngology procedures (thyroidectomy, parotidectomy, hypoglossal nerve stimulator implantation, drug‐induced sleep endoscopy, septoplasty, or tonsillectomy) from 2014 to 2022. OOP costs were defined as the sum of deductibles, copays, and coinsurance for each 30‐day surgical episode. A potential surprise bill was defined as an out‐of‐network claim within an episode where both the primary surgeon and facility were in‐network. Relationships between OOP costs, potential surprise bills, and patient‐ and system‐level exposures were analyzed.

**Results:**

Of 58,772 procedures meeting inclusion criteria, 52,131 (89%) procedures were outpatient and 6641 (11%) were inpatient. Median (interquartile range [IQR]) total OOP costs were $1207 ($183‐$2594), and coinsurance accounted for 66% of OOP costs. OOP costs were higher for patients with insurance plans that were fee‐for‐service‐based (2.9 times; *P* < .001) or high‐deductible (4.7 times; *P* < .001) versus managed care plans. A potential surprise bill accompanied 4.8% of surgical encounters, which was associated with significantly higher OOP costs (median $1739 vs $1269; *P* < .001). The odds of having a potential surprise bill were lower in 2022 versus 2014 to 2021 (odds ratio [OR] 0.29, 95% CI 0.21‐0.40; *P* < .001).

**Conclusion:**

Common elective otolaryngology procedures were associated with high OOP expenditures, potentially exceeding the reserve funds of many patients. Potential surprise bills decreased after the passage of the No Surprises Act but were not eliminated.

Out‐of‐pocket (OOP) healthcare costs are a major contributor to bankruptcy in the United States and are linked to poor health outcomes, including delayed care, medication nonadherence, lower quality of life, and reduced survival rates.^1^, ^2^, ^3^, ^4^, ^5^, ^6^, ^7^, ^8^, ^9^, ^10^ In 2023, an estimated 27% of adults in the United States went without medical care because they could not afford it, and 42% of adults were unable to afford more than $1000 in emergency expenses using savings.^11^ Surgery accounts for up to half of healthcare spending in the United States,^12^ and OOP costs associated with surgery plausibly exceed reserve funds for many patients.^13^, ^14^ However, few data are available regarding costs of elective surgery in otolaryngology or whether OOP costs have changed amid recent policy reforms.

OOP costs for surgery can be difficult for patients to predict due to cost‐sharing measures such as coinsurance,^14^ which are often poorly understood by the public.^15^ Additionally, patients may be subject to “surprise” bills, which are out‐of‐network charges that occur despite having a primary surgeon and facility within their insurance network.^16^ Legislation including the Hospital Price Transparency Rule and No Surprises Act went into effect in 2021 and 2022, respectively, aiming to address these issues.^17^, ^18^, ^19^ The Hospital Price Transparency Rule requires hospitals to publish consumer‐friendly OOP cost estimates for specified “shoppable” services.^18^ The No Surprises Act purports that patients cannot be billed more for out‐of‐network services than they would for the same service with an in‐network provider.^19^ However, little is known about whether these policies proved effective, in part because of gaps in the policies that warrant further evaluation. For example, the Hospital Price Transparency Rule does not apply to freestanding ambulatory surgery centers and has suboptimal compliance rates.^20^, ^21^ The No Surprises Act defers to individual states to determine fair out‐of‐network rates, which could undermine federal standards.

A better understanding of OOP costs and surprise billing in otolaryngology, especially in the context of recent legislation, is necessary for understanding the experiences and unintended consequences of patients undergoing otolaryngologic surgery. This could ultimately help guide patient counseling and inform interventions aims at improving the affordability of surgical care. To help address this gap, we used the Merative MarketScan database to evaluate OOP costs and potential surprise bills for patients undergoing six common otolaryngology procedures from 2014 to 2022. Based on literature for other surgical specialties, we hypothesized that the median OOP costs would exceed $1000 per surgical episode and would be driven primarily by coinsurance. We also hypothesized that procedures involving neuromonitoring or neurostimulation would be more likely to have a potential surprise bill and that potential surprise bills would decrease in frequency after implementation of the No Surprises Act.

## Methods

This study was reviewed by the University of Michigan Institutional Review Board and deemed not regulated on the grounds that it used a de‐identified publicly available data set (IRB#: HUM00227012).

### Data Source and Sample

We identified individual claims from the Merative MarketScan Commercial Database.^22^ The MarketScan database contains de‐identified billing data for more than 250 million patients with commercial insurance plans in the United States from 2007 to 2022. It represents all 50 states and approximately 350 private, employer‐sponsored payers.

We included patients if they underwent one of the six procedures of interest between 2014 and 2022 and were between 18 and 64 years old at the time of surgery. The six procedures were selected as a sampling of operations within the scope of comprehensive otolaryngology. The procedures are performed across different practice settings (eg, academic vs nonacademic hospitals, inpatient vs outpatient), and allowed testing of our hypotheses related to neurostimulation/neuromonitoring. Patients receiving surgery before 2014 were excluded, given that the Inspire® device (Inspire Medical Systems, Inc.) for hypoglossal nerve stimulation (HGNS) had not yet been approved by the Food and Drug Administration. Patients older than 64 years were excluded because Medicare and Medicare Advantage plans prohibited balance billing throughout the study period. Supplemental File S1, available online, provides further details regarding the cohort selection.

### Primary Outcomes and Exposure Variables

Our primary outcomes were OOP costs and potential surprise bills. OOP costs were defined as the sum of coinsurance, deductibles, and copays for a 30‐day surgical episode. A potential surprise bill was defined as an out‐of‐network claim for a surgical episode where both the primary surgeon and facility were classified as in‐network. Network status was based on plan definitions. This variable was provided by MarketScan and coded as a dichotomous variable with inputs of either “yes” or “no” for each claim, based on whether the service was in‐network or out‐of‐network. This information was provided directly to MarketScan by the insurance companies. Procedures potentially involving neuromonitoring/neurostimulation were defined as thyroidectomy, parotidectomy, and HGNS implantation. Procedures unlikely to involve neuromonitoring/neurostimulation were drug induced sleep endoscopy (DISE), septoplasty, and tonsillectomy. Insurance plans were categorized as fee‐for‐service, high‐deductible health plan (HDHP), or managed care plan, consistent with other studies in the current literature.^23^ Fee‐for‐service plans were plans without capitation, including basic/major medical, comprehensive, exclusive provider organization, point‐of‐service plan, preferred provider organization, or consumer‐driven health plans. HDHPs were classified in MarketScan according to the deductible and contribution limits indexed each year by the United States Treasury Department. Managed care plans included fully or partially capitated plans, including both health maintenance organization (HMO) plans and capitated point‐of‐service plans. Year of surgery was categorized as 2022 versus 2014 to 2021 to facilitate hypothesis testing related to the timing of the *No Surprises Act*.

### Statistical Analysis

All OOP costs were adjusted for inflation based on 2024 dollars using the consumer price index.^24^ Cost data were winsorized to the 1st and 99th percentiles to account for the effects of outliers. The distributions of the cost data were right‐skewed, so for the subsequent analyses, we reported median and interquartile range (IQR) and used nonparametric testing. Wilcoxon rank sum tests were used to compare OOP costs based on inpatient/outpatient status, facility type, and neuromonitoring/neurostimulation classification. We used generalized linear regression models on log‐transformed cost data to evaluate associations with total OOP costs. Back‐transformation was used to obtain cost ratios, which represent the multiplicative change in total OOP costs relative to the reference exposure group.

We used chi‐square tests to evaluate differences in the prevalence of potential surprise bills by inpatient/outpatient status, facility type, and neuromonitoring/neurostimulation classification. Logistic regression models were used to evaluate associations with having a potential surprise bill. We used two a priori adjustment sets for the models for both OOP costs and surprise billing (one adjusted for procedure type and another adjusted for both procedure type and inpatient/outpatient status) to account for potential confounding. We used SAS software version 9.4 for all analyses, and significance was set at *P* < .05.

## Results

### Patient Sample

A total of 58,772 patients were included in the sample with a mean (SD) age of 41.5 (13.9) years. Fifty‐eight percent of patients were female, and most patients (79.3%) had a fee‐for‐service‐based insurance plan. There were 17,882 procedures potentially involving neuromonitoring/neurostimulation (thyroidectomy, parotidectomy, and HGNS implantation) and 40,890 procedures without neuromonitoring/neurostimulation (DISE, septoplasty, and tonsillectomy). A total of 11,378 (19.4%) of surgeries were performed in the hospital setting, and 6641 surgeries (11%) were classified as inpatient. Baseline characteristics are summarized in Table 1.

**Table 1:** Baseline Characteristics

Variable	Number (%) or mean (SD)
Age (mean, SD), y	41.5 (13.9)
Sex	
Female	34,068 (58.0%)
Male	24,704 (42.0%)
Year	
2014‐2021	56,030 (95.3%)
2022	2742 (4.7%)
Region	
Northeast	10,662 (18.1%)
North Central	9668 (16.5%)
South	27,618 (47.0%)
West	10,449 (17.8%)
Unknown	375 (0.6%)
Insurance type	
Fee‐for‐service plan	45,561 (79.3%)
High‐deductible health plan (HDHP)	4387 (7.6%)
Managed care plan	7525 (13.1%)
Place of service	
Outpatient hospital on campus	40,517 (68.9%)
Ambulatory surgery center	15,106 (25.7%)
Inpatient hospital	11,378 (19.4%)
Other	1770 (3.0%)
Employment status	
Actively employed	40,517 (68.9%)
Retired	3149 (5.4%)
Other/unknown	15,106 (25.7%)

### OOP Costs

**Figure 1:**
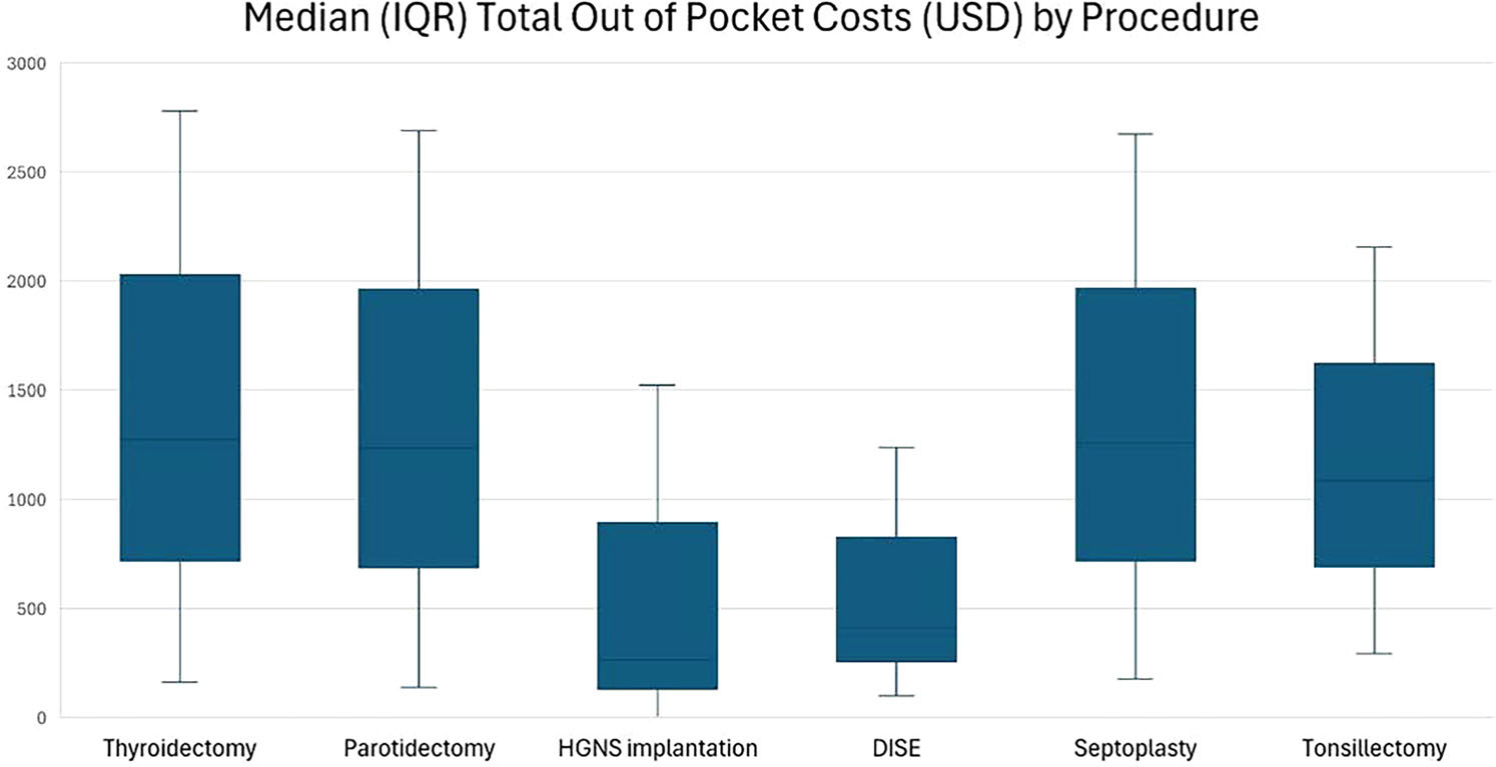
Median total out‐of‐pocket costs by procedure. DISE, drug induced sleep endoscopy; HGNS, hypoglossal nerve stimulation; IQR, interquartile range.

The median (IQR) total OOP costs across all procedures and study years were $1207 ($183‐$2594). The OOP costs stratified by procedure are depicted in Table 2 and Figure 1. Median total OOP costs were highest for thyroidectomy ($1274) and lowest for HGNS implantation ($263). Mean (SD) total OOP costs were $1707 ($1837) across all procedures; mean (SD) coinsurance was $1133 (SD 1358); mean (SD) deductible was $490 (SD $999); mean (SD) copay was $68 (SD $141). Coinsurance was the largest contributor to OOPs across all procedures, accounting for 66.4% of mean total costs. Total OOP costs were significantly lower for inpatient surgeries compared to outpatient surgeries (median [IQR] $961 [93‐2764] vs $1231 [195‐2578]; *P* < .001). When stratified by outpatient status, there were no significant differences in total OOP costs at outpatient hospitals compared to ambulatory surgery centers (median [IQR] $1271 [178‐2636] vs $1183 [297‐2416]; *P* = .785).

**Table 2:** Median (Interquartile Range [IQR]) Out‐of‐Pocket Costs

Procedure	N	Total	Coinsurance	Deductible	Copay
Thyroidectomy	14,883	1274 (161‐2779)	808 (0‐2170)	0 (0‐288)	0 (0‐79)
Parotidectomy	2539	1235 (137‐2689)	782 (0‐2147)	0 (0‐212)	0 (0‐66)
HGNS implantation	460	263 (0‐1523)	248 (0‐2028)	0 (0‐104)	0 (0‐51)
DISE	305	414 (99‐1239)	128 (0‐601)	0 (0‐326)	0 (0‐88)
Septoplasty	29,612	1257 (177‐2674)	797 (0‐1887)	0 (0‐560)	0 (0‐87)
Tonsillectomy	10,973	1086 (294‐2155)	517 (0‐1183)	0 (0‐858)	0 (0‐61)

Abbreviations: DISE, drug induced sleep endoscopy; HGNS, hypoglossal nerve stimulation.

For outpatient surgeries, surgeries potentially involving neuromonitoring/neurostimulation had significantly higher OOP costs than surgeries without neuromonitoring/neurostimulation (median [IQR] $1378 [194‐2774] vs $1193 [195‐2502]; *P* < .001). However, for inpatient surgeries, surgeries potentially involving neuromonitoring/neurostimulation had significantly lower OOP costs (median [IQR] $891 [75‐2645] vs $1273 [197‐$3230]; *P* < .001).

Significant predictors of total OOP costs, adjusted for procedure type and inpatient/outpatient status, are summarized in Table 3. Older age and an employment status of “retired” were associated with significantly lower OOP costs (*P* < .001 for both). Significant predictors of higher OOP costs included year 2022 (vs 2014‐2021; *P* = .007), region of North Central, South, or West (vs Northeast; *P* < .001 for all), and fee‐for‐service plan or HDHP (vs managed care plan; *P* < .001 for both).

**Table 3:** Predictors of Total Out‐of‐Pocket Costs

	Cost ratio and 95% CI^a^	*P*‐value	Cost ratio and 95% CI^b^	*P*‐value
Age (mean, SD), per 10‐y increase	0.95 (0.94‐0.96)	<.001	0.95 (0.94‐0.96)	<.001
Sex		.331		.206
Female	Reference		Reference	
Male	1.01 (0.99‐1.04)		1.02 (0.99‐1.04)	
Year		.007		.007
2014‐2021	Reference		Reference	
2022	1.08 (1.02‐1.15)		1.08 (1.02‐1.14)	
Region				
Northeast	Reference		Reference	
North Central	2.22 (2.13‐2.31)	<.001	2.22 (2.13‐2.31)	<.001
South	2.58 (2.49‐2.66)	<.001	2.58 (2.50‐2.67)	<.001
West	1.66 (1.59‐1.72)	<.001	1.66 (1.59‐1.72)	<.001
Unknown	1.30 (1.12‐1.51)	<.001	1.30 (1.13‐1.51)	<.001
Insurance type				
Fee‐for‐service plan	2.85 (2.75‐2.95)	<.001	2.85 (2.75‐2.95)	<.001
High‐deductible health plan (HDHP)	4.71 (4.47‐4.97)	<.001	4.71 (4.47‐4.97)	<.001
Managed care plan	Reference		Reference	
Employment status				
Actively employed	Reference		Reference	
Retired	0.68 (0.64‐0.72)	<.001	0.68 (0.64‐0.72)	<.001
Other/unknown	1.07 (1.04‐1.10)	<.001	1.07 (1.04‐1.10)	<.001

^a^ Adjusted for procedure type (thyroidectomy, parotidectomy, hypoglossal nerve stimulation implantation, drug induced sleep endoscopy, septoplasty, and tonsillectomy).

^b^ Adjusted for procedure type and inpatient/outpatient.

### Surprise Billing

**Figure 2:**
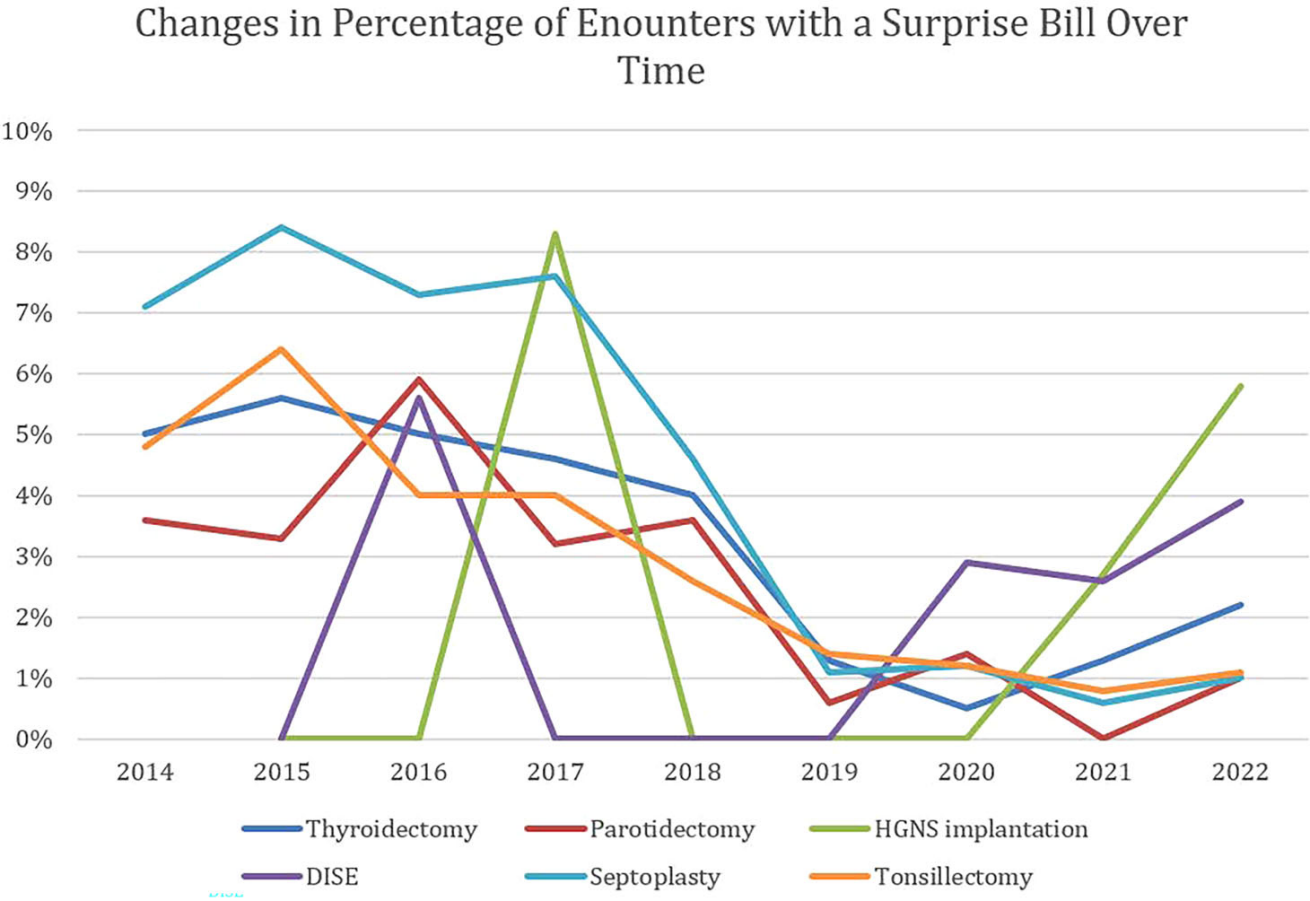
Changes in prevalence of surprise bills over time by procedure. DISE, drug induced sleep endoscopy; HGNS, hypoglossal nerve stimulation.

A total of 57,026 surgical episodes had sufficient data for the surprise billing analysis, and among these, 2624 had a potential surprise bill (4.8%). Table 4 and Figure 2 depict the prevalence of potential surprise bills stratified by procedure and year. The prevalence of potential surprise bills was similar for inpatient and outpatient procedures (5.3% vs 4.8%; *P* = .059). When stratified by outpatient status, there was a lower prevalence of potential surprise bills at outpatient hospitals compared to ambulatory surgery centers (4.2% vs 6.4%; *P* < .001).

**Table 4:** Prevalence of Potential Surprise Bills Stratified by Procedure and Year

	2014	2015	2016	2017	2018	2019	2020	2021	2022
Thyroidectomy, %	5.0	5.6	5.0	4.6	4.0	1.3	0.5	1.3	2.2
Parotidectomy, %	3.6	3.3	5.9	3.2	3.6	0.6	1.4	0	1.0
HGNS implantation, %	n/a	0	0	8.3	0	0	0	2.7	5.8
DISE, %	n/a	0	5.6	0	0	0	2.9	2.6	3.9
Septoplasty, %	7.1	8.4	7.3	7.6	4.6	1.1	1.2	0.6	1.0
Tonsillectomy, %	4.8	6.4	4.0	4.0	2.6	1.4	1.2	0.8	1.1
Overall, %	6.0	7.2	6.0	6.0	4.0	1.1	1.0	0.9	1.5

Abbreviations: DISE, drug induced sleep endoscopy; HGNS, hypoglossal nerve stimulation.

Neuromonitoring/neurostimulation procedures had a lower prevalence of potential surprise bills compared to procedures not involving neurostimulation/neuromonitoring (3.9% vs 5.2%; *P* < .001). Surgical episodes with a potential surprise bill had significantly higher total OOP costs compared to those without (median [IQR] $1739 [365‐3630] vs $1269 [198‐2619]; *P* < .001).

Significant predictors of a potential surprise bill, adjusted for procedure type and inpatient/outpatient status, are summarized in Table 5. Significant predictors of having a potential surprise bill included older age (*P* < .001), a fee‐for‐service plan, or HDHP (vs managed care plan; *P* < .001 for both), and an employment status of “retired” (*P* < .001). Year 2022 (vs 2014‐2021; *P* < .001), region of North Central, South, or West (vs Northeast; *P* < .001 for all), and neuromonitoring/neurostimulation procedure (*P* < .001) were associated with lower odds of having a potential surprise bill.

**Table 5:** Predictors of Surprise Billing

	Odds ratio and 95% CI^a^	*P*‐value	Odds ratio and 95% CI^b^	*P*‐value
Age (y), per 10‐y increase	1.08 (1.04‐1.11)	<.001	1.08 (1.04‐1.11)	<.001
Sex				
Female	Reference		Reference	
Male	1.07 (0.99‐1.15)	.072	1.06 (0.98‐1.13)	.127
Year				
2014‐2021	Reference		Reference	
2022	0.29 (0.21‐0.40)	<.001	0.29 (0.21‐0.40)	<.001
Region				
Northeast	Reference		Reference	
North Central	0.58 (0.52‐0.64)	<.001	0.58 (0.53‐0.65)	<.001
South	0.42 (0.38‐0.45)	<.001	0.42 (0.39‐0.46)	<.001
West	0.37 (0.33‐0.42)	<.001	0.37 (0.33‐0.42)	<.001
Unknown	0.98 (0.70‐1.39)	.927	0.99 (0.70‐1.39)	.938
Insurance type				
Fee‐for‐service plan	2.68 (2.31‐3.12)	<.001	2.69 (2.31‐3.13)	<.001
High‐deductible health plan (HDHP)	2.52 (2.08‐3.05)	<.001	2.52 (2.08‐3.05)	<.001
Managed care plan	Reference		Reference	
Employment status				
Actively employed	Reference		Reference	
Retired	1.36 (1.17‐1.59)	<.001	1.37 (1.17‐1.59)	<.001
Other/unknown	0.55 (0.50‐0.62)	<.001	0.55 (0.49‐0.61)	<.001
Potential use of neurostimulation/neuromonitoring				
Neurostimulation	0.43 (0.38‐0.48)	<.001	0.54 (0.47‐0.62)	<.001
Non‐neurostimulation	Reference		Reference	

^a^ Adjusted for procedure type (thyroidectomy, parotidectomy, hypoglossal nerve stimulation implantation, drug induced sleep endoscopy, septoplasty, and tonsillectomy).

^b^ Adjusted for procedure type and inpatient/outpatient.

When analyzing the claims‐level data, 82,225 of 1,523,684 claims (5.4%) were classified as a potential surprise bill. Most claims that were classified as a potential surprise bill were professional claims (n = 68,174; 82.9%). The top three sources of professional claims classified as potential surprise bills were otolaryngology (n = 51,470; 63.0%), surgeon “not elsewhere classified” (n = 5846; 7.2%), and plastic/maxillofacial surgery (n = 2133; 2.6%). Physician assistants and nurse practitioners accounted for only 417 (0.51%) and 28 (0.03%), respectively. Medical technicians accounted for 152 (0.19%) of claims classified as potential surprise bills.

## Discussion

Our comprehensive analysis of 58,772 common otolaryngology procedures found median OOP costs of $1207 across surgeries. Furthermore, coinsurance, which is often poorly understood by patients,^15^ accounted for 66% of total OOP costs. Potential surprise bills accompanied 4.8% of surgical encounters and were associated with higher OOP costs, likely contributing to unexpected medical bills for patients. We also identified insurance plan type as a major driver of cost, which can inform efforts to improve the affordability of surgical care. These findings lend insights into OOP costs in relation to recent legislation.

Median OOP costs have been reported to exceed $1000 across a variety of surgeries, including bariatric surgery, hysterectomy, lumbar discectomy, nephrectomy, colectomy, and total knee replacement.^13^, ^14^, ^25^, ^26^ Our findings align with studies in other surgical fields that have identified coinsurance as the largest contributor to OOP surgical costs.^14^, ^23^, ^27^ A comprehension test by Loewenstein et al estimated that only 34% of Americans understand the concept of coinsurance,^15^ likely contributing to higher than anticipated medical costs for many individuals undergoing surgery. Taken together, these findings suggest that common elective otolaryngology procedures may exceed the financial reserves of many Americans with commercial insurance, and the degree of financial burden may not be apparent on the front end.

Facility type (outpatient hospital vs ambulatory care center) is another potentially modifiable factor for patients receiving outpatient otolaryngologic surgery. Prior studies have identified higher facility fees for otolaryngologic surgery at outpatient hospitals compared to ambulatory care centers,^28^ which may be attributable to differences in infrastructure costs and market power between facilities. Our analysis was reassuring for no significant differences in total OOP costs faced by patients between facility types. It is possible that insurance payments could be serving as a buffer to protect patients from differences in facility costs. For example, a study evaluating outpatient hand surgeries found that ambulatory surgery centers were associated with approximately $1000 lower facility costs but only about $50 lower OOP costs compared to outpatient hospitals, suggesting that only a small fraction of the cost difference is felt by the patient.^29^

Potential surprise bills decreased in frequency over the study period, consistent with our hypothesis related to the timing of the *No Surprises Act*. Patients with a fee‐for‐service‐based plan or HDHP had the highest risk of a potential surprise bill, with adjusted odds ratios of 2.69 and 2.52, respectively, compared to managed care plans. To our knowledge, no other studies have evaluated surprise billing in otolaryngology, but our findings are consistent with the literature from other surgical specialties. A MarketScan database study of patients undergoing hysterectomy from 2008 to 2018 found that the overall prevalence of surprise billing was 8.8%; surprise bills decreased in frequency over the study period and were associated with older age, higher comorbidity burden, and surgical complications.^30^ Another study of patients undergoing seven elective surgical operations from 2012 to 2017 found a higher rate of potential surprise bills (20%), though these data came from a different payer source.^16^ Differences between our findings and those from other studies using data from a single payor could reflect differences in insurance network coverage beyond the primary surgeon and facility.

Insurance type appears to be a significant driver of OOP costs and potential surprise billing for patients undergoing common otolaryngology procedures. While fee‐for‐service plans and HDHPs may offer expanded network access and lower premiums, they appear to afford less protection against cost sharing and surprise billing. HDHPs have become increasingly popular in the past two decades,^31^ and they have been identified as a primary driver of OOP costs in surgery across several studies.^26^, ^27^, ^32^, ^33^, ^34^ Preoperative discussion about expected OOP costs and optimization of insurance plan could help mitigate some of these problems. Studies in plastic surgery have found that 50 to 70 percent of patients undergoing breast reconstruction desire a preoperative discussion about costs with their surgeon.^35^, ^36^ In oncology, financial navigator programs have been used to help optimize patients' insurance plans before treatment and help identify cost‐reduction strategies.^37^, ^38^ The potential role for financial navigators in otolaryngology clinics is a promising area for future research. Even in the absence of dedicated navigators, preoperative provision of information by the healthcare system regarding OOP costs with their insurance plan can enhance transparency and informed decisions for patients undergoing surgery.

Our finding that potential surprise bills were less likely in procedures with possible neurostimulation or neuromonitoring was unexpected. A study in orthopedic surgery found that 28% of surprise bills for lumbar discectomy were attributable to neurologists, presumably through the use of third‐party contractors for intraoperative neuromonitoring.^39^ The use of third‐party contractors for neuromonitoring in procedures such as HGNS implantation, thyroidectomy, and parotidectomy (eg, neurophysiologists or audiologists) is likely more variable than for lumbar discectomy, which could partially account for these differences in findings. Additionally, data from the orthopedic surgery study were derived from a single insurer through the Clinformatics database,^39^ which could have implications on provider networks and the risk of surprise bills. Finally, in contrast to other databases, the Marketscan database did not have a specific code for neuromonitoring or neurostimulation, so we had to use the type of procedure as an indirect proxy to estimate the possibility of neuromonitoring/neurostimulation. Therefore, the findings regarding neurostimulation/neuromonitoring should be interpreted with caution.

When stratified by procedure, both OOP costs and the prevalence of potential surprise bills appear to be lower for DISE and HGNS compared to the other procedures. Lower OOP costs for DISE and HGNS may be related to comprehensive insurance coverage for these procedures if patients meet the preoperative requirements for age, body mass index, apnea‐hypopnea index, and CPAP intolerance. In addition, DISE and HGNS could have lower OOP costs if preceded by prior treatments that lead patients to reach their deductible for the year. These explanations are consistent with a previous analysis finding mean cumulative OOP costs of approximately $300 for DISE and HGNS despite hospital and insurance costs exceeding $40,000.^40^ The lower prevalence of potential surprise bills observed for DISE and HGNS could reflect that these procedures are less likely than other procedures to be accompanied by use of independent laboratories or pathologists, services which are a common source of surprise billing.^41^ Future work using databases with more granular data on surprise billing could help in elucidating these findings.

We found that the prevalence of potential surprise billing was highest in the Northeast, consistent with other studies using different databases.^42^ Additional literature demonstrates that the prevalence of surprise billing tends to be lowest in the Midwest but has high state‐to‐state level variability.^16^, ^41^ Interestingly, these studies indicate that the prevalence of surprise billing was not necessarily any lower in states that had legal protections against surprise billing before implementation of the No Surprises Act (CA, CT, FL, IL, CO, MA, MD, MS, NJ, NY, OR, NH, and AZ).^16^, ^41^ These findings suggest that insurance market concentration and provider networks may account for some of the geographic differences in surprise billing. Individual plans purchased on the health insurance exchange increase the risk of potential surprise bills.^16^, ^41^ Health insurance exchange plans have narrower provider networks, and this problem is magnified in metropolitan areas,^43^ which could partially account for the higher prevalence of surprise billing observed in the Northeast.

There was an increase in the prevalence of potential surprise bills in 2022 from the prior 3 years (difference of up to 0.6%), although the prevalence of 1.5% remained well below the peak in prior years. While the significance of this increase is uncertain, the end of the COVID‐19 pandemic and the pent‐up demand for elective surgery that followed it could be a factor. The process of expediting backlogged cases in 2022 may have taken precedence over optimizing insurance coverage and network status before surgery. Future studies with data after 2022 may help to contextualize this finding.

The recent presidential guidance on the Hospital Price Transparency Rule is notable,^44^ and the relationship between this legislation and the prevalence of surprise billing is an area for further exploration. In theory, improving preoperative price transparency could reduce the incidence of surprise billing for elective surgeries if the price displayed to the patient accurately reflects the patient's insurance plan and network status. However, relevance for otolaryngology may be limited currently because pediatric tonsillectomy and adenoidectomy are the only otolaryngologic procedures listed among the 70 “shoppable” services that hospitals are required to include.^17^, ^18^

Our study has several limitations. First, we are unable to estimate whether the potential surprise bill was actually sent to the patient, which is a limitation ubiquitous across studies evaluating surprise billing with claims data. The MarketScan database primarily captures patients with commercial health insurance, so our findings are not generalizable to patients with Medicaid and Medicare. Furthermore, the database does not provide socioeconomic data, which is an important context for understanding the burden of OOP costs. We are unable to assess whether the OOP costs identified in our study exceeded the reserve funds for patients in our sample; estimates of reserve funds were based on prior literature. Similarly, we were unable to measure how much of the costs faced by patients were anticipated versus unexpected, which could have implications for financial well‐being and quality of life. There are also several potential unmeasured confounders that could influence the relationship between our exposure variables and OOP costs or surprise bills, including patient comorbidities, operative time, hospital length of stay, and surgical complications. Last, the procedures studied represent only a small subset of surgery in otolaryngology, and the findings may not generalize to the overall specialty. Despite these limitations, our study provides novel data on OOP costs and surprise billing in otolaryngology and is consistent with other data in surgical fields, supporting their validity. The findings highlight the need for future studies with more granular patient socioeconomic data, measures of indirect costs, qualitative perspectives on financial toxicity related to surgery, and representation of insurance plans outside of the commercial market.

## Conclusions

OOP costs for the sample of otolaryngology procedures studied may exceed the reserve funds for some patients. OOP costs are likely difficult to anticipate due to contributions from coinsurance and surprise billing. Insurance plan type appears to be the greatest modifiable factor for OOP costs and surprise billing.

## Author Contributions

**Nicholas R. Lenze**, conceptualization, methodology, formal analysis, writing—original draft, visualization, funding acquisition; **Chamila D. Perera**, methodology, software, formal analysis, resources, data curation, writing—review and editing; **Karan R. Chhabra**, conceptualization, methodology, resources, writing—review and editing; **John W. Scott**, conceptualization, methodology, resources, writing—review and editing; **Raj C. Dedhia**, conceptualization, methodology, resources, writing—review and editing; **Michael J. Brenner**, conceptualization, methodology, resources, writing—review and editing, supervision.

## Disclosures

### Competing interests

The authors declare no conflicts of interest.

### Funding source

Nicholas R. Lenze was supported by the Michigan Otolaryngology Research Education (MORE) NIH R25 research grant (DC020262) and the Centralized Otolaryngology Research Efforts (CORE) grant for this project. Karan R. Chhabra is supported by NYU CTSI grant KL2 TR001446 from the National Center for Advancing Translational Sciences, National Institutes of Health.

## Supporting information

Supporting Information.
