# Supplementary material for: Out‐of‐Pocket Costs and Surprise Billing in Otolaryngology: A National Database Analysis
Source: Otolaryngol Head Neck Surg. 2025 Oct 13;173(6):1393–402. doi: 10.1002/ohn.70035 (PMC12661487; doi:10.1002/ohn.70035)
Supplement: Supplementary file 1 — Supporting Information. [file OHN-173-1393-s001.docx]

Supplemental File 1

Cohort Selection

To obtain our cohort, we first queried the MarketScan inpatient and outpatient databases for relevant claims using the CPT codes for our procedures of interest, including thyroidectomy, parotidectomy, hypoglossal nerve stimulator implantation, drug induced sleep endoscopy (DISE), septoplasty, or tonsillectomy (**Table S1**). For CPT codes pertaining to drug-induced sleep endoscopy or HGNS, we limited inclusion to patients with a concomitant diagnosis code for obstructive sleep apnea (OSA) to ensure specificity, and we thereby excluded unrelated flexible laryngoscopy procedures or other types of nerve implants that were not HGNS. Additionally, we only included DISE claims if they also had a claim for HGNS on a later date.

Next, we limited our cohort to patients with continuous enrollment in their insurance plan for at least 3 months before and at least 3 months after the day of surgery. Only claims with coinsurance, deductibles, or copays greater than or equal to $0 were included. To capture all potentially relevant claims, we defined a surgical episode to include all claims from the date of surgery to 30 days after surgery. Any surgical episodes without at least one professional claim and one facility claim were excluded. We aggregated claims data into patient-specific, encounter-level data based on the 30-day surgical episode definition. For patients who had more than one eligible procedure during the study period, we selected the procedure at the earliest date.

**Table S1:** CPT Codes of Interest

| **Procedure** | **Specifics** | **CPT Code** | **Modifiers or other codes** |
| --- | --- | --- | --- |
| **Thyroidectomy** |  |  |  |
|  | Total | 60240 |  |
|  | Partial/lobectomy | 60220 |  |
|  | hemithyroid, partial contralateral | 60225 |  |
|  | Completion/revision thyroidectomy | 60260 |  |
|  | Total or subtotal with limited neck dissection | 60252 |  |
|  | Total or subtotal with radial neck dissection | 60254 |  |
|  | Total including substernal thyroidectomy with sternal split or transthoracic approach | 60270 |  |
|  | Thyroidectomy including substernal thyroid, cervical approach | 60271 |  |
|  | Excision of thyroid cyst or adenoma | 60200 |  |
|  | Partial lobectomy | 60210 |  |
|  | Partial lobectomy with contralateral subtotal lobectomy | 60212 |  |
| **Parotidectomy** |  |  |  |
|  | Excision of parotid tumor or parotid gland; lateral lobe, without nerve dissection | 42410 |  |
|  | Excision of parotid tumor or parotid gland; lateral lobe, with dissection and preservation of facial nerve | 42415 |  |
|  | Excision of parotid tumor or parotid gland; total, with dissection and preservation of facial nerve | 42420 |  |
|  | Excision of parotid tumor or parotid gland; total, en bloc removal with sacrifice of facial nerve | 42425 |  |
|  | Excision of parotid tumor or parotid gland; total, with unilateral radical neck dissection | 42426 |  |
| **HGNS implantation*** |  |  | *only included if patients have a diagnosis of obstructive sleep apnea (G47.33) |
|  | implantation of cranial nerve (e.g., vagus nerve) neurostimulator electrode array and pulse generator | 64568 | +0466T (modifier for chest wall sensor electrode) |
|  | Open implantation of hypoglossal nerve neurostimulator array, pulse generator and distal respiratory sensor electrode or electrode array | 64582 (after 1/1/2022) |  |
| **DISE*** |  |  | *only include if patients have a diagnosis of obstructive sleep apnea (G47.33) |
|  | Endoscopy Procedures on the Larynx | 31575 |  |
|  | Drug Induced Sleep Endoscopy (DISE) | 42975 (after 1/1/2022) |  |
| **Septoplasty** |  |  |  |
|  | septoplasty | 30520 |  |
| **Tonsillectomy** |  |  |  |
|  | Tonsillectomy, primary or secondary, patient 12 years or older | 42826 |  |
